# A young man with out-of-hospital cardiac arrest—it goes round and round

**DOI:** 10.1007/s12471-020-01481-3

**Published:** 2020-07-31

**Authors:** S. C. M. D. Panman, J. M. ter Maaten, Y. Blaauw

**Affiliations:** Department of Cardiology, University Medical Center Groningen, University of Groningen, Groningen, The Netherlands

## Answer

The electrocardiogram (ECG) at presentation at the emergency department showed a sinus rhythm of 98 beats per minute with a delta wave. ECG findings are suggestive of pre-excitation with an accessory pathway between the atria and ventricle, as has been described by Wolff, Parkinson and White. Using the Arruda algorithm, the localisation of the accessory pathway is most likely left posterior [1].

The ECG during palpitations at the cardiac care unit showed an atrioventricular reciprocating tachycardia (AVRT), a macroreentrant tachycardia over an accessory pathway, the most common type of arrhythmia associated with the Wolff-Parkinson-White syndrome [2]. An AVRT can start after an extra atrial or ventricular beat over the slow pathway when the fast pathway is still refractory, with retrograde conduction over the accessory pathway. Treatment of choice is a sodium channel blocker that blocks conduction across and prolongs the refractoriness of the accessory pathway, allowing the fast pathway to take over [3].

**Fig. 1:**
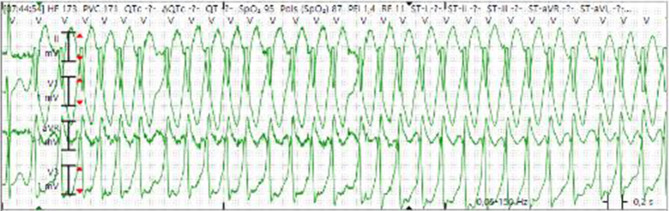
Fast, broad and irregular tachycardia

**Fig. 2:**
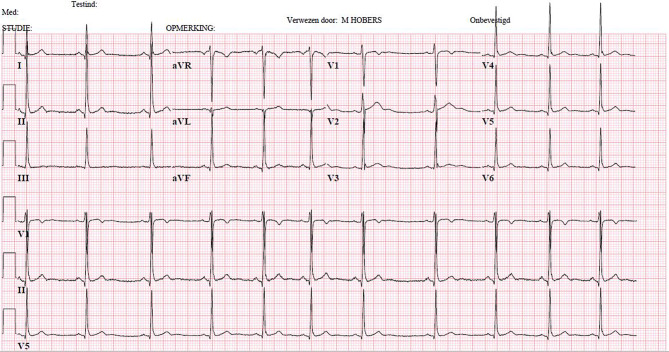
The electrocardiogram after ablation of a left posterior pathway noting the absence of a delta wave

During the first 12 h after admission our patient had recurrent AVRT episodes. We treated him with flecainide after which the AVRT episodes ended almost every time. One time it changed into atrial fibrillation with antidromic conduction over the accessory pathway, also known as FBI—fast, broad and irregular—(Fig. 1), requiring acute cardioversion. Given the delta wave on the ECG, the high recurrence rate of supraventricular tachycardia episodes, and the out-of-hospital cardiac arrest, the day after admission an electrophysiology study (EPS) was performed. Indeed, a left posterior accessory pathway was identified and successfully ablated. Fig. 2 shows the ECG after pathway ablation, where no delta wave was visible any longer. Therefore, when there is a high clinical suspicion of a Wolff-Parkinson-White syndrome, EPS should be considered.
